# Phase I study of daily and weekly regimens of the orally administered MDM2 antagonist idasanutlin in patients with advanced tumors

**DOI:** 10.1007/s10637-021-01141-2

**Published:** 2021-06-28

**Authors:** Antoine Italiano, Wilson H. Miller, Jean-Yves Blay, Jourik A. Gietema, Yung-Jue Bang, Linda R. Mileshkin, Hal W. Hirte, Brian Higgins, Steven Blotner, Gwen L. Nichols, Lin Chi Chen, Claire Petry, Qi Joy Yang, Christophe Schmitt, Candice Jamois, Lillian L. Siu

**Affiliations:** 1 Institut Bergonié, Bordeaux, France; 2 Gustave Roussy, Villejuif, France; 3 Faculty of Medicine, University of Bordeaux, Bordeaux, France; 4 Segal Cancer Centre, Jewish General Hospital, McGill University, Montreal, QC Canada; 5 Centre Léon Bérard, Lyon, France; 6 University Medical Center Groningen, University of Groningen, Groningen, the Netherlands; 7 Seoul National University College of Medicine, Seoul, South Korea; 8 Peter MacCallum Cancer Center, Melbourne, Australia; 9 Juravinski Cancer Centre, Hamilton, ON Canada; 10 Roche Innovation Center, Hoffmann-La Roche, New York, NY USA; 11 Roche Innovation Center, Basel, Switzerland; 12 Certara, Montréal, QC Canada; 13 Princess Margaret Cancer Centre, Toronto, ON Canada

**Keywords:** p53 activation, MDM2 antagonist, Cis-imidazoline analog, Idasanutlin, Nutlin, MIC-1

## Abstract

**Summary:**

*Aim* The oral MDM2 antagonist idasanutlin inhibits the p53-MDM2 interaction, enabling p53 activation, tumor growth inhibition, and increased survival in xenograft models. *Methods* We conducted a Phase I study of idasanutlin (microprecipitate bulk powder formulation) to determine the maximum tolerated dose (MTD), safety, pharmacokinetics, pharmacodynamics, food effect, and clinical activity in patients with advanced malignancies. Schedules investigated were once weekly for 3 weeks (QW × 3), once daily for 3 days (QD × 3), or QD × 5 every 28 days. We also analyzed p53 activation and the anti-proliferative effects of idasanutlin. *Results* The dose-escalation phase included 85 patients (QW × 3, n = 36; QD × 3, n = 15; QD × 5, n = 34). Daily MTD was 3200 mg (QW × 3), 1000 mg (QD × 3), and 500 mg (QD × 5). Most common adverse events were diarrhea, nausea/vomiting, decreased appetite, and thrombocytopenia. Dose-limiting toxicities were nausea/vomiting and myelosuppression; myelosuppression was more frequent with QD dosing and associated with pharmacokinetic exposure. Idasanutlin exposure was approximately dose proportional at low doses, but less than dose proportional at > 600 mg. Although inter-patient variability in exposure was high with all regimens, cumulative idasanutlin exposure over the whole 28-day cycle was greatest with a QD × 5 regimen. No major food effect on pharmacokinetic exposure occurred. MIC-1 levels were higher with QD dosing, increasing in an exposure-dependent manner. Best response was stable disease in 30.6% of patients, prolonged (> 600 days) in 2 patients with sarcoma. *Conclusions* Idasanutlin demonstrated dose- and schedule-dependent p53 activation with durable disease stabilization in some patients. Based on these findings, the QD × 5 schedule was selected for further development.

**Trial registration:**

NCT01462175 (ClinicalTrials.gov), October 31, 2011.

**Supplementary information:**

The online version contains supplementary material available at 10.1007/s10637-021-01141-2.

## Introduction

The p53 tumor suppressor protein is a powerful pro-apoptotic factor that plays a central role in inhibiting tumor development [1]. MDM2, a negative regulator of the p53 tumor suppressor, functions as an E3 ubiquitin ligase, targeting p53 for proteasomal degradation [2]. A class of imidazoline compounds, termed “Nutlins,” has been identified as potent and selective inhibitors of the p53–MDM2 interaction, interacting specifically with the p53 binding pocket of MDM2 and thus releasing p53 from negative control [3–5]. Using Nutlins to treat cancer cells that express functional p53 stabilizes p53 and activates its pathway, leading to activation of p53-target genes, cell cycle arrest, apoptosis, and/or senescence [4]. RG7112, the first Nutlin compound to enter the clinic, established clinical proof of mechanism in *MDM2*-amplified liposarcoma [6, 7]. Further, response per Response Evaluation Criteria in Solid Tumors (RECIST) was achieved in previous RG7112 clinical trials. However, patients had difficulty tolerating treatment on the requisite daily RG7112 schedule [6, 8].

Idasanutlin (RG7388, RO5503781) is a second-generation selective inhibitor of the p53–MDM2 interaction. Idasanutlin retains the in vitro anti-tumor activity of RG7112 but has superior pharmacologic characteristics, including improved potency, bioavailability, and selectivity for the p53 binding site of MDM2 [5, 9–15]. In preclinical modeling and simulation studies, both daily and intermittent dosing with idasanutlin were predicted to be effective at achieving tumor stasis [10, 11]. Thus, despite the relatively short half-lives of p53 and MDM2, continuous suppression of the p53–MDM2 interaction did not appear to be required for optimal antitumor activity. These preclinical data suggested that intermittent idasanutlin dosing could provide the same activity as daily dosing.

The preclinical evidence together with the improved pharmacological properties of idasanutlin supported the rationale for shorter intermittent dosing schedules in this initial Phase I study, with the aim of decreasing the risk of thrombocytopenia. The aim of this first-in-human study was to investigate the maximum tolerated dose (MTD), characterize dose-limiting toxicity (DLT), and explore the safety, tolerability, pharmacokinetics (PK), pharmacodynamics (PD), and clinical responses with idasanutlin with different dosing schedules.

## Materials and methods

### Study design and treatment

**Fig. 1:**
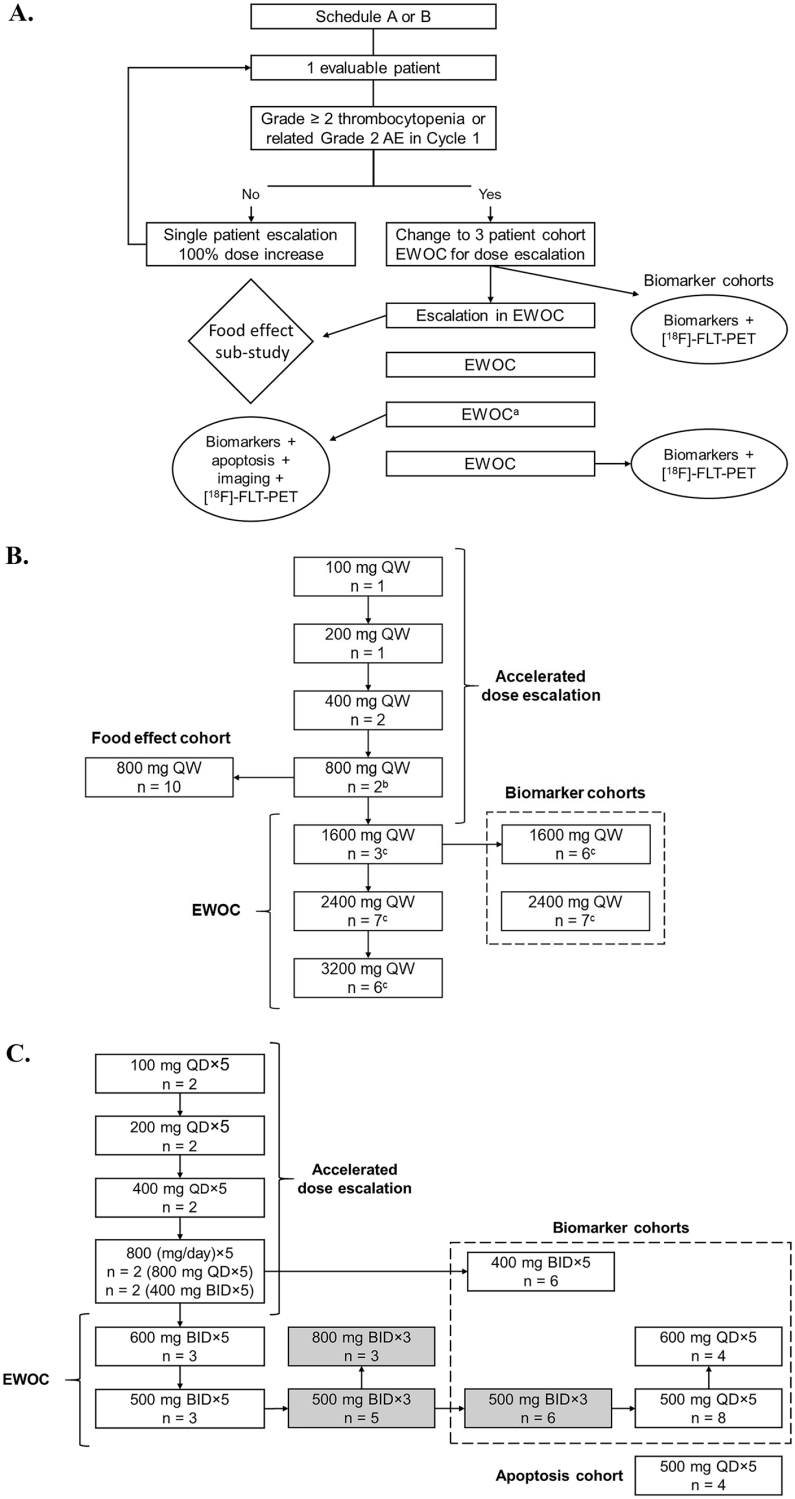
Study design and treatment allocation schedules. **A** Study overview. **B** Treatment allocation for QW × 3 schedule (schedule A). **C** Treatment allocation for QD × 3 and QD × 5 schedules (schedule B). For the weekly regimen (schedule A), idasanutlin was administered orally at a total daily dose of 100–3200 mg once weekly for 3 weeks followed by 13 days of rest. For the daily regimens (schedule B), idasanutlin was administered orally at a total daily dose of 1000 mg or 1600 mg daily for 3 days followed by 25 days of rest, or at a total daily dose of 100–1200 mg daily for 5 days followed by 23 days of rest. Arrows indicate the sequence of opening the cohorts (panels **B** and **C**). Gray boxes indicate cohorts with 3 planned dosing days per cycle only. AE, adverse event; BID, twice daily; EWOC, escalation with overdose control; QD, once daily; QW, once weekly. ^a^ Not all EWOC cohorts had associated biomarker evaluations. ^b^One patient was dosed BID. ^c^Patients were dosed BID

This was a multicenter, open-label, Phase I, multiple ascending dose-escalation trial of single-agent idasanutlin in a microprecipitate bulk powder (MBP) formulation in patients with advanced malignancies other than leukemia (ClinicalTrials.gov identifier: NCT01462175). Following the accelerated dose-escalation phase using an escalation with overdose control (EWOC) design [16], additional patients were recruited to food-effect and apoptosis-imaging cohorts (only safety data reported; Fig. 1). The dose-escalation phase involved single-patient cohorts until grade 2 related adverse events (AE) were reported (Fig. 1). Based on these AE criteria, subsequent dose escalation involved 3-patient cohorts in a modified continual-reassessment-method EWOC design. Idasanutlin was administered either once daily (QD; (to define abbreviation at first use of term) when total daily dose was at or below 800 mg) or twice daily (when total daily dose was at or exceeded 800 mg). For the weekly regimen, idasanutlin was administered orally at a total daily dose of 100–3200 mg once weekly (QW) for 3 weeks (QW × 3) followed by 13 days of rest. For the daily regimens, idasanutlin was administered orally at a total daily dose of 1000 mg or 1600 mg QD for 3 days (QD × 3) followed by 25 days of rest, or at a total daily dose of 100–1200 mg daily for 5 days (QD × 5) followed by 23 days of rest. Idasanutlin was given without food, except in the food effect sub-study. Treatment continued until disease progression, unacceptable toxicity, or withdrawal of consent.

### Patients

Eligible patients were at least 18 years of age with histologically confirmed advanced malignancies (except any type of leukemia) for which standard curative or palliative measures were unavailable, no longer effective, or unacceptable to the patient. All patients were required to have measurable disease per RECIST 1.1 criteria (or Cheson criteria for malignant lymphomas) and Eastern Cooperative Oncology Group performance status of 0 to 1. There were no limitations on additional type or amount of prior anti-tumor therapy. Acute toxicities from any prior anti-cancer therapy, surgery, or radiotherapy must have resolved to grade ≤ 1 per National Cancer Institute Common Terminology Criteria for Adverse Events v4.03. Patients had to have adequate bone marrow function, renal function (serum creatinine within normal limits or creatinine clearance ≥ 50 mL/min [calculated by Cockcroft-Gault equation]), and hepatic function (with serum total bilirubin ≤ 2 mg/dL). Those with stable central nervous system metastases or chronic, stable, and rate-controlled atrial fibrillation were eligible. To be eligible for the biomarker cohort, patients had to have a tumor that could be biopsied prior to and during treatment. Exclusion criteria included use of other investigational drugs ≤ 4 weeks prior to study treatment start; preexisting gastrointestinal disorders that could interfere with drug absorption; and history of seizure disorders or unstable central nervous system metastases.

### Endpoints and assessments

The primary study objectives were to: determine the MTD of different idasanutlin dosing schedules administered in 28-day cycles to fasted patients; characterize the DLTs and overall safety profile across escalated dose levels; and explore these dosing schedules for safety and tolerability. Secondary objectives included determining the PK and PD profiles of idasanutlin, as well as clinical responses.

The safety population, defined as all patients who received ≥ 1 dose of the study drug, was used for all safety, efficacy, and PK analyses. The DLT-evaluable population comprised patients who completed the first 28-day treatment cycle (i.e., received ≥ 4 days of therapy in the QD × 5 schedule or ≥ 2 days in the QW × 3 schedule) and had sufficient safety evaluations. AEs were reported according to the Medical Dictionary for Regulatory Activities, version 17.0, with severity graded by National Cancer Institute Common Terminology Criteria for Adverse Events v4.03.

Efficacy-evaluable patients included those in the safety population with a baseline assessment and ≥ 1 post-baseline tumor assessment. Tumor response (objective response rate) was assessed approximately every 8 weeks using RECIST 1.1 criteria for patients with measurable or non-measurable lesions or using Cheson criteria for those with malignant lymphomas.

Further details on the assessments of the food effect, PK (Online Resource Table S1), PD, and biomarkers are available in the Supplementary Methods in the Online Resource.

## Results

### Patient population

A total of 99 patients were enrolled and treated between November 15, 2011, and July 4, 2014. Dose-escalation cohorts included those on the QW × 3 (n = 36), QD × 3 (n = 15), and QD × 5 (n = 34) regimens; 10 and 4 patients were included in the food-effect and apoptosis-imaging cohorts, respectively (Fig. 1). Thirty-seven patients from the dose-escalation phase were included in the biomarker analysis cohorts (QW × 3, n = 13; QD regimens, n = 24).

Baseline characteristics were similar across cohorts; the median age was 57.0 years (range, 23–76 years; Online Resource Table S2). More than two-thirds of patients (69.4%) had an Eastern Cooperative Oncology Group performance status of 1. All patients had been treated with ≥ 1 prior anti-cancer regimen, with the majority (70.7%) having received 1 to 4 regimens. The most common tumor types were sarcoma (n = 23 [23.2%]) and colorectal cancer (n = 12 [12.1%]). *TP53* mutations were analyzed at baseline (archival and pre-dose tumor samples) and, where possible, in paired pre- and post-biopsy samples. Mutations in *TP53* were detected in 19 of 58 archival samples (32.8%) and 12 of 32 pre-dose biopsies (37.5%) (Online Resource Table S2).

### Safety and tolerability

The median duration of treatment for all patients was 36 days (range, 1–726 days), with 15 patients (15.2%) treated for > 91 days (Online Resource Table S3). The median (range) number of total daily doses received in the QW × 3, QD × 3, and QD × 5 cohorts, respectively, was 10.5 (2–72), 9.0 (6–42), and 10.0 (1–130). All 99 patients comprised the safety population; across all cohorts, 78 patients (78.8%) received ≤ 2 treatment cycles.

The MTD for QW × 3 dosing was 3200 mg (given as 1600 mg twice daily [BID]), with DLTs of nausea, thrombocytopenia, and vomiting (Online Resource Table S4), all reported at a total daily dose of 1600 mg or higher. The MTD for QD × 3 dosing was 1000 mg (given as 500 mg BID), with DLTs of thrombocytopenia, febrile neutropenia, neutropenia, and pancytopenia. For QD × 5 dosing, the MTD was 500 mg (given QD), with DLTs of thrombocytopenia, neutropenia, febrile neutropenia, and diarrhea. A total of 31 DLTs were reported across all cohorts (n = 99), with 21 patients (21.2%; dose-escalation cohorts, n = 20; apoptosis cohort, n = 1) having ≥ 1 DLT. The most common DLT was thrombocytopenia, occurring in 16 of 99 patients (16.2%). Other DLTs included neutropenia (5 [5.1%]), febrile neutropenia (3 [3.0%]), nausea (2 [2.0%]), as well as leukopenia, pancytopenia, diarrhea, and vomiting (1 each [1.0%]). Within the dose-escalation cohorts, DLTs were more common in patients on daily (40% for QD × 3 and 32.4% for QD × 5) versus weekly dosing schedules (8.3%), with a higher incidence of DLTs related to hematologic and lymphatic system disorders reported with daily regimens (Online Resource Table S4).

All 99 patients experienced ≥ 1 AE that was considered by the investigator to be related to study treatment (Table 1). The most common treatment-related AEs were diarrhea (74.7%), nausea (71.7%), vomiting (50.5%), decreased appetite (43.4%), and thrombocytopenia (39.4%; Online Resource Table S5). In general, treatment-related AEs occurred at the highest frequencies with the QD × 3 schedule; the lowest frequencies were observed with the QD × 5 schedule (Online Resource Table S5).

**Table 1:** Overview of AEs

	Weekly dosing (QW × 3)^a^ (n = 36)	Daily dosing^b^		Food effect cohort (n = 10)	Apoptosis cohort (n = 4)	Total (N = 99)
		QD × 3 (n = 15)	QD × 5 (n = 34)			
Total no. of events						
SAEs, n	10	12	27	2	2	53
DLT AEs, n	4	8	17	0	2	31
Deaths (due to fatal AE or PD), n (%)	2 (5.6)	0	4 (11.8)	1 (10.0)	0	7 (7.1)
No. of patients with ≥ 1 event, n (%)						
AE	36 (100.0)	15 (100.0)	34 (100.0)	10 (100.0)	4 (100.0)	99 (100.0)
Related AE	36 (100.0)	15 (100.0)	34 (100.0)	10 (100.0)	4 (100.0)	99 (100.0)
Grade ≥ 3 AE	21 (58.3)	11 (73.5)	25 (73.5)	4 (40.0)	2 (50.0)	63 (63.6)
SAE	9 (25.0)	7 (46.7)	13 (38.2)	2 (20.0)	1 (25.0)	32 (32.3)
Related SAE	4 (11.1)	7 (46.7)	13 (38.2)	0	1 (25.0)	25 (25.3)
DLT AE	3 (8.3)	6 (40.0)	11 (32.4)	0	1 (25.0)	21 (21.2)
AE with fatal outcome	1 (2.8)	0	1 (2.9)	0	0	2 (20.0)
AE leading to treatment withdrawal	4 (11.1)	3 (20.0)	10 (29.4)	1 (10.0)	0	18 (18.2)
AE leading to dose modification/interruption	16 (44.4)	7 (46.7)	18 (52.9)	2 (20.0)	1 (25.0)	44 (44.4)

*AE* adverse event, *DLT* dose-limiting toxicity, *PD* progressive disease, *QD* once daily, *QW* once weekly, *SAE* serious adverse event

^a^Weekly dosing schedule, including biomarker cohorts, except those enrolled in the food effect sub-study

^b^Daily dosing schedules (QD × 3 or QD × 5), including biomarker cohorts, except those enrolled in the apoptosis cohort

Grade ≥ 3 AEs of any cause occurred in 63 patients (63.6%) and were reported in higher incidences in the QD dosing regimens (Table 1). The most common grade ≥ 3 any-cause AEs were thrombocytopenia (29.3%), anemia (20.2%), neutropenia (16.2%), nausea (11.1%), and diarrhea (7.1%). Serious AEs (SAEs) were reported in 32 patients (32.3%) across all study groups (Table 1; Online Resource Table S6). Treatment-related SAEs were reported in 25 patients (25.3%); the most frequently reported (in 24 of 25 patients) were related to blood and lymphatic system disorders: thrombocytopenia/decreased platelet count (14 events), febrile neutropenia (5 events), neutropenia/decreased neutrophil count (4 events), leukopenia/decreased white blood cell count (3 events), and anemia (2 events). Treatment-related SAEs were more frequently reported with QD dosing (QD × 3, 7 of 15 [46.7%]; QD × 5, 13 of 34 [38.2%]) than QW × 3 dosing (4 of 36 [11.1%]) (Online Resource Table S6).

The majority of patients (81 of 99 [81.8%]) discontinued treatment due to non-safety reasons: disease progression (n = 77), patient consent withdrawal (n = 3), and other reason unspecified (n = 1). Eighteen patients (18.2%) withdrew due to AEs, 8 of which were considered SAEs. More AE-related discontinuations occurred in patients receiving daily dosing regimens, excluding the apoptosis imaging cohort (QD × 3, 20.0%; QD × 5, 29.4%), compared with those receiving QW × 3, excluding the food effect cohort (11.1%). The most common AEs resulting in study drug discontinuation among all patients were neutropenia, thrombocytopenia, and pulmonary embolism (3.0% each). AEs associated with study withdrawal were more likely to be hematologic in nature and grade ≥ 3 in severity.

Dose modifications/interruptions due to an AE were reported in 44 patients (44.4%) and occurred at similar frequencies with the weekly and daily schedules (Table 1). This included 16 of 36 patients (44.4%) on QW × 3 dosing, while 7 of 15 patients (46.7%) on QD × 3 dosing and 18 of 34 (52.9%) on QD × 5 dosing required dose modifications. The most frequently reported AEs leading to dose modification were thrombocytopenia (24.2%) and neutropenia (9.1%).

Overall, 7 deaths occurred during treatment or over the 28 days following the last study dose: 5 were due to progressive disease and 2 were due to an SAE (Table 1). One death (QW × 3 cohort) was due to an intra-abdominal hemorrhage and pulmonary embolism, both determined to be unrelated to study treatment. The other death (QD × 5 cohort) was due to pulmonary embolism and possibly related to study treatment.

### Clinical activity

Of the 85 response-evaluable patients, none achieved an objective response, and 26 patients (30.6%) had a best overall response of stable disease (SD). Of the 26 patients with SD, 7 had modest tumor shrinkage not approaching objective response (-1, -4, -7, -8, -8, -9, and -11%). Rates of SD were comparable among patients receiving weekly dosing (10 of 33 patients; 30.3%) and daily dosing (QD × 3, 5 of 13 [38.5%]; QD × 5, 8 of 26 [30.8%]). Median overall duration of SD was 72.5 days (range, 8–696 days); this was shorter in the QD × 3 cohort (57.0 days) than in the QW × 3 or QD × 5 cohorts (99.0 and 103.0 days, respectively). For patients who were treated > 3 cycles, the duration of idasanutlin exposure is shown in Online Resource Fig. S1.

Ten patients (7 with sarcoma, 1 with melanoma, 1 with testicular cancer, and 1 with mesothelioma) experienced SD for > 100 days. The 2 patients with the longest duration of SD (> 600 days) were on the QD × 5 schedule; both had sarcomas (Online Resource Fig. S1). One of these patients, who received 200 mg idasanutlin QD × 5 and had a non-functional (deleterious) *TP53* R158H mutant leiomyosarcoma, achieved SD for 620 days. The other patient, who received 800 mg idasanutlin QD × 5 and had a *TP53* wild-type extra-skeletal myxoid chondrosarcoma, achieved SD for 696 days.

### Pharmacokinetics

**Fig. 2:**
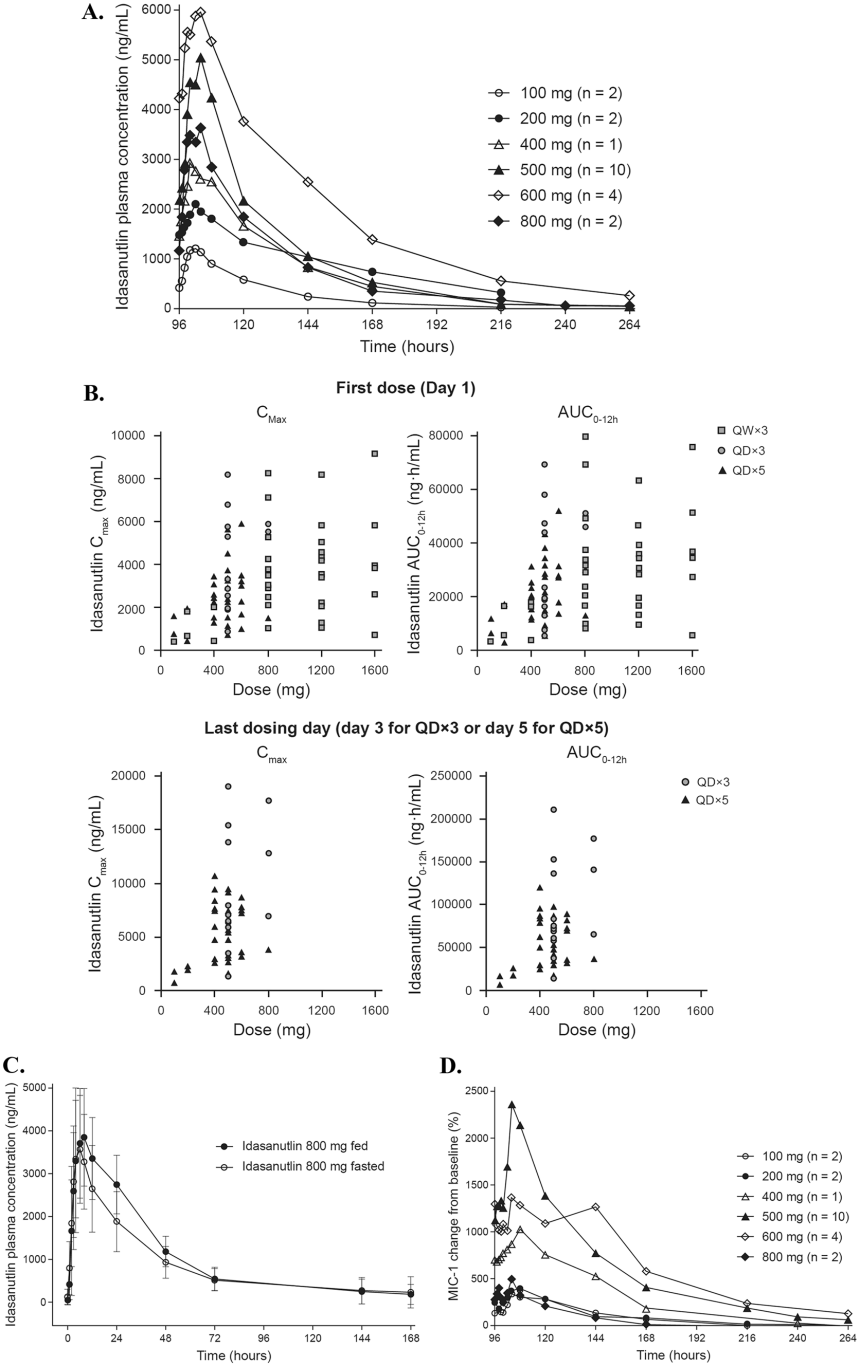
Idasanutlin pharmacokinetics and pharmacodynamics. **A** Mean plasma concentration–time profiles of idasanutlin following oral administration of once daily dose for 5 days; data for last dose on day 5 (dose escalation, biomarker, and apoptosis cohort). **B** Scatterplots of idasanutlin exposure versus administered dose on first and last days of idasanutlin treatment. Data for last dosing day do not include patients who received QW × 3 dosing. **C** Plasma concentration–time profiles of idasanutlin following oral administration of a single 800-mg dose in the fasted and fed state. Data are presented as arithmetic mean values (95% confidence interval); n = 10. **D** Mean serum concentration–time profiles of MIC-1 following oral administration of once daily dose of idasanutlin for 5 days; data for last dose on day 5 (dose escalation, biomarker, and apoptosis cohort). Arithmetic means. AUC_0-12 h_, area under curve from 0 to 12 h; C_max_, maximum plasma concentration; QD, once daily; QW, once weekly

Idasanutlin peak concentrations typically occurred 6 to 8 h after oral administration without food, and declined thereafter, with terminal half-life of ≈30 h (Fig. 2A). Exposure was approximately dose proportional after the first dose (i.e., day 1) and following repeat dosing (i.e., day 3 for QD × 3 regimens, day 5 for QD × 5 regimens), although increases appeared to be less than dose proportional at doses above 600 mg, suggesting a saturation in intestinal absorption at this dose level (Fig. 2B). However, inter-patient variability in exposure was high with all dosing regimens (Fig. 2B). Exposure was approximately twofold higher on the final day of QD × 3 and QD × 5 dosing compared with the first dose (Fig. 2B), but there was no accumulation with QW × 3 dosing (data not shown). For a specified daily dose, cumulative idasanutlin exposure over the whole 28-day dosing cycle was greatest with a QD × 5 regimen, reflecting the higher total dose administered (i.e., 5 days of dosing vs. 3 days of dosing or 3 single doses).

### Food effects on idasanutlin pharmacokinetics

Ten patients received 800-mg doses of idasanutlin, either with a high-fat/high-calorie meal or while fasted. Dosing employed a half-replicate, crossover design, which resulted in 15 pairs of fed versus fasted data from the 10 patients. On average, idasanutlin exposure was higher when taken with food (mean maximum plasma concentration was 14% higher and area under the curve extrapolated to infinity was 43% higher), but variability was high and as the 90% confidence intervals encompassed unity, it was concluded that food had no clinically meaningful effect on idasanutlin exposure (Online Resource Table S7; Fig. 2C).

### Pharmacodynamic analysis

After idasanutlin dosing, circulating macrophage inhibitory cytokine 1 (MIC-1) levels increased generally in a dose-exposure–dependent manner (Fig. 2D). Consequently, trends in MIC-1 responses to treatment mirrored trends in idasanutlin exposure, as described in Population PK/PD Analysis section.

Sixteen of the 31 patients (51.6%) evaluated by positron emission tomography analysis for changes in tumor proliferation rates with idasanutlin treatment achieved a partial proliferative response as their best percentage maximum standardized uptake value change from baseline during cycle 1, indicating a decrease of ≥ 25% (Online Resource Fig. S2).

### Population PK/PD analysis

**Fig. 3:**
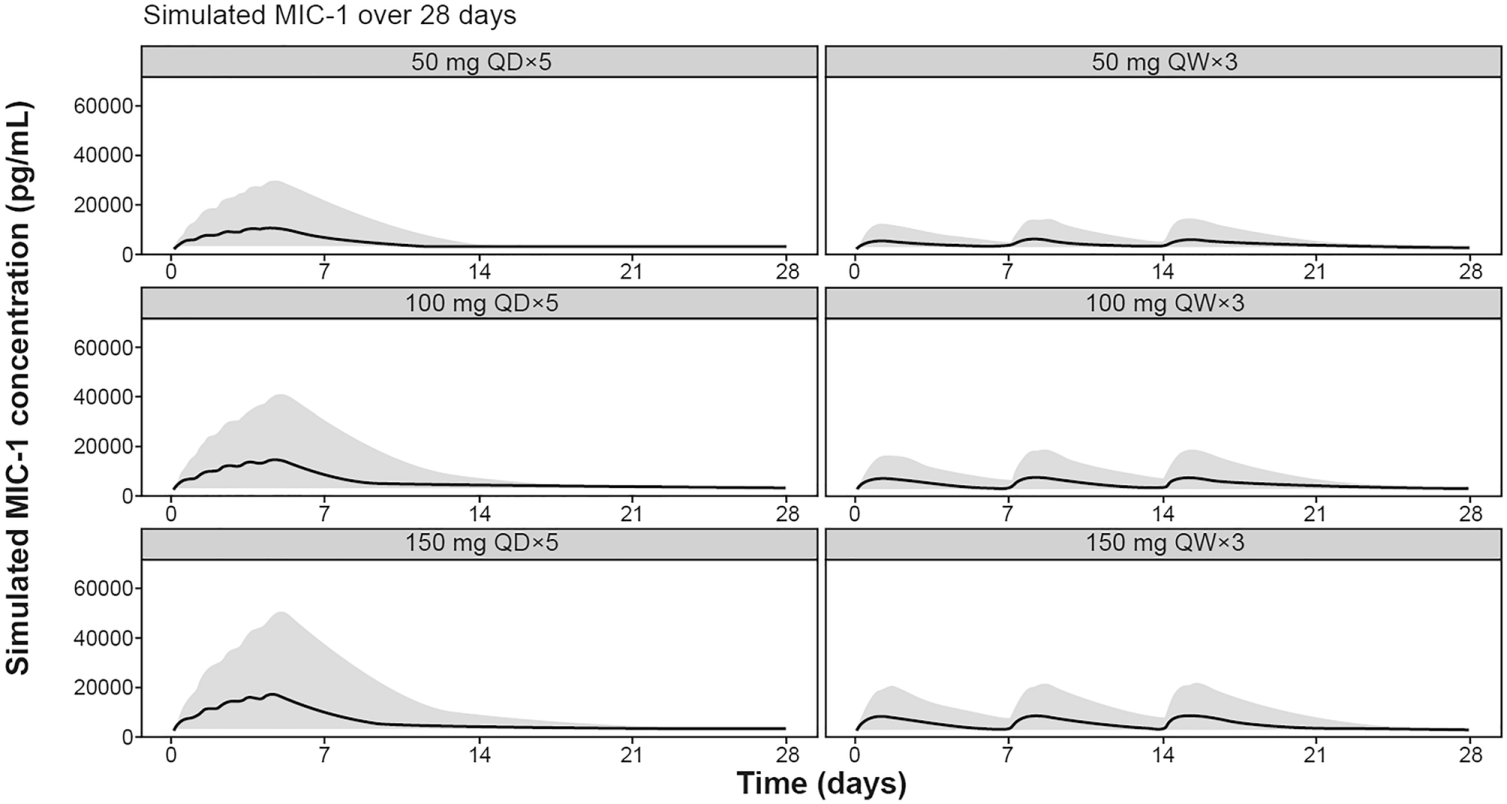
Simulated MIC-1 release over 28 days following QD or QW regimen. The black line is the median of the predicted MIC-1 concentrations; the gray area is the 90% prediction interval around the median. Results are simulations with the indirect PK/MIC-1 model (stimulation of MIC-1 release). MIC-1, macrophage inhibitory cytokine 1; QD, once daily; QW, once weekly

Simulations with the indirect PK/MIC-1 model (Online Resource Supplementary Methods) indicated that despite some high variability, the release of MIC-1 following idasanutlin treatment is concentration dependent; the higher the idasanutlin concentrations, the higher the release of MIC-1. Weekly dosing with idasanutlin resulted in lower maximum release but a more sustainable effect on MIC-1 over the 28-day treatment cycle compared with a daily regimen (for the same level of dose) (Fig. 3).

**Fig. 4:**
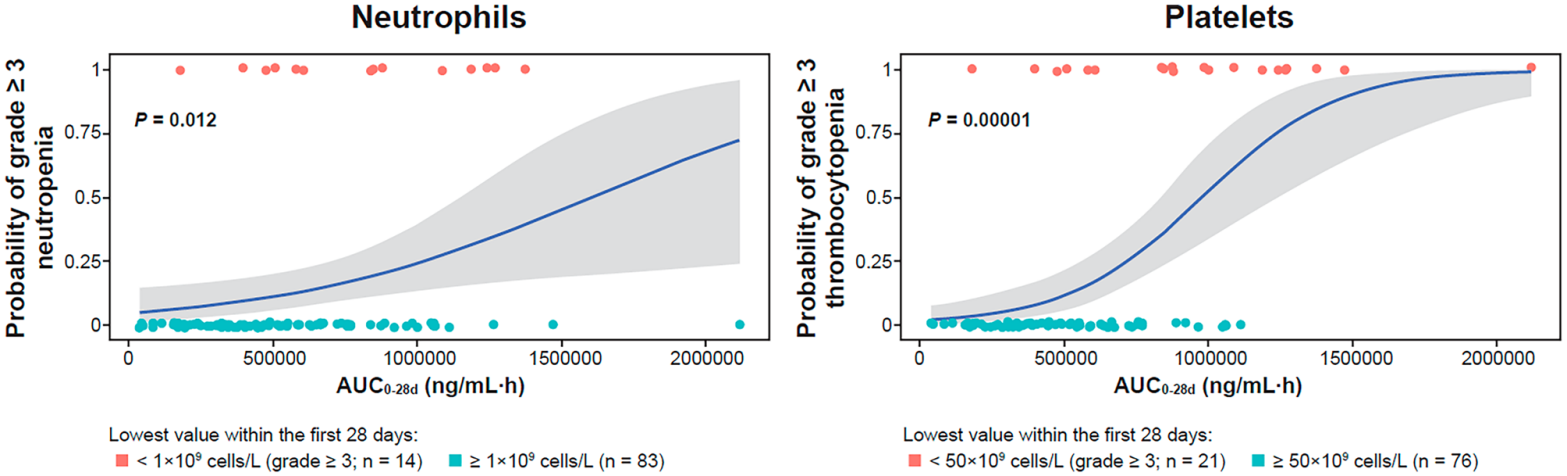
Relationship between idasanutlin exposure and probability of occurrence of grade ≥ 3 neutropenia and grade ≥ 3 thrombocytopenia during cycle 1. Idasanutlin exposure is the cumulative area under the curve over 28 days (AUC_0-28d_) in ng/mL·h (individual exposures were derived using a population PK module developed on data from different disease types [see Supplementary Methods]). Neutropenia grade ≥ 3 events are the lowest absolute neutrophil count value < 1 × 10^9^ cells/L with 28 days of treatment. Thrombocytopenia grade ≥ 3 events are the lowest platelet count < 50 × 10^9^ cells/L with 28 days of treatment

The risk of grade ≥ 3 neutropenia and thrombocytopenia events was exposure dependent as shown in Fig. 4. The higher the exposure over 28 days, the higher the risk of occurrence of events. Due to its lower exposure, the weekly dosing is predicted to have lower risk of neutropenia or thrombocytopenia compared with a daily regimen for the same dose level.

## Discussion

The MTD for weekly dosing of idasanutlin MBP was identified as 3200 mg, while for daily dosing, it was 500 mg (QD × 5 dosing) and 1000 mg (QD × 3 dosing). DLTs were primarily hematologic and gastrointestinal for all schedules. The MTD for daily dosing was much lower than that for weekly dosing, and as a result, a larger proportion of patients were treated at doses close to or above the MTD on the daily dosing schedule. The dose-escalation pattern resulted in more-frequent treatment-related AEs or higher-intensity AEs, grade ≥ 3 AEs, and SAEs with the daily schedule than with weekly dosing. The use of supportive therapies, including empiric anti-emetic prophylaxis and growth factor support, was able to manage the AEs.

The AE profile observed with idasanutlin was consistent with those in previous studies of MDM2 antagonists [6, 8]. In solid tumors, hematologic effects must be considered after idasanutlin treatment, especially in combination therapy. Besides receiving supportive care for AE management (such as growth factor support to treat leukopenia and neutropenia, and transfusions of packed red blood cells to support anemia), patients must be monitored carefully for thrombocytopenia developing in the latter part of a dosing cycle or for neutropenia.

Idasanutlin’s PK profile was characterized by high inter-patient variability, which confounded quantitative comparisons between dosing regimens. Overall, cumulative idasanutlin exposure over the whole 28-day dosing cycle depended on the dosing regimen used and was greatest with a QD × 5 regimen. The higher exposure was also reflected in a greater PD effect (Fig. 2D). No clinically meaningful food effect on idasanutlin exposure was observed. Consequently, induction of p53 as measured by MIC-1 levels occurred as a function of idasanutlin levels and treatment schedule, and trends in MIC-1 responses mirrored trends in idasanutlin exposure. MIC-1 induction was greater with daily than weekly dosing schedules, indicating that a daily schedule results in greater p53 activation than the weekly schedule.

PK/PD analyses also suggested an apparent relationship between idasanutlin exposure and hematologic toxicity. There was, however, no clearly identifiable difference in the idasanutlin exposure–hematological toxicity relationships between dosing regimens, suggesting that the apparent differences in tolerability between daily and weekly dosing can be attributed to differences in total idasanutlin exposure.

Population PK/PD modeling and simulations predict that, for the same dose level, a weekly dosing regimen will be associated with lower (as measured by MIC-1), although more sustained, target engagement over a 28-day treatment cycle and a lower risk of grade ≥ 3 myelosuppressive events than a 5-day regimen. For the same cumulative dose, the PK/PD analyses did not suggest differentiation between the weekly and 5-day daily dosing regimens in target engagement or risk of grade ≥ 3 myelosuppressive events. Exposure–response relationships based on monotherapy data predict limited target engagement and minimal risk of myelosuppression at idasanutlin doses currently being tested in combination studies.

Objective anti-tumor responses with idasanutlin (by RECIST 1.1 or Cheson criteria) were not observed in this study. Median durations of SD tended to be longer with the QW × 3 and QD × 5 schedules than with the QD × 3 schedule. Any associations of efficacy with *TP53* mutation status or any specific mutations in *TP53* cannot be made in the absence of objective clinical responses. The MTD on the daily dosing schedule for patients with solid tumors (500 mg for QD × 5 or 1000 mg for QD × 3) is significantly lower than the 600-mg BID dose in the MBP formulation used in patients with relapsed/refractory acute myeloid leukemia in whom hematologic effects are a desired outcome [17]. It is reasonable to hypothesize that in patients with solid tumors, a dose higher than the MTD on either daily dosing schedule is required for monotherapy efficacy, or that combination therapy will need to be explored.

A comparison of MIC-1 elevation between the idasanutlin dosing schedules demonstrated that the daily schedule resulted in greater p53 activation than the weekly schedule. The QD × 3 regimen, however, did not achieve steady-state exposure and did not avert thrombocytopenia. Therefore, the QD × 5 schedule was selected for subsequent clinical trials based on the supportive evidence of p53 activation, comparable safety profiles of the QD × 3 and QD × 5 regimens, and ability to manage AEs with supportive therapies.

## Conclusion

Idasanutlin led to exposure-dependent p53 activation with durable disease stabilization in select patients; no clinically meaningful food effect was observed. The daily QD × 5 schedule was identified for further clinical study, although alternative dosing schedules may be considered depending on the toxicity profile and/or combination partner. Idasanutlin is now being administered in an optimized spray dried powder formulation with approximately twofold higher bioavailability compared with the MBP formulation.

## Supplementary information

Below is the link to the electronic supplementary material.

Supplementary file1 (DOCX 221 KB)

Supplementary file2 (DOCX 41 KB)

## Data Availability

Qualified researchers may request access to individual patient level data through the clinical study data request platform (https://vivli.org/). Further details on Roche's criteria for eligible studies are available here (https://vivli.org/members/ourmembers/). For further details on Roche's Global Policy on the Sharing of Clinical Information and how to request access to related clinical study documents, see here (https://www.roche.com/research_and_development/who_we_are_how_we_work/clinical_trials/our_commitment_to_data_sharing.htm).
