# Supplementary material for: Phase I study of daily and weekly regimens of the orally administered MDM2 antagonist idasanutlin in patients with advanced tumors
Source: Invest New Drugs. 2021 Jun 28;39(6):1587–97. doi: 10.1007/s10637-021-01141-2 (PMC8541972; doi:10.1007/s10637-021-01141-2)

**SUPPLEMENTARY FIGURES**

**Fig. S1** Days of idasanutlin exposure in patients treated for > 3 cycles. ^a^ *TP53* mutant; ^b^ *TP53* wild type; ^c^ *TP53* unknown.


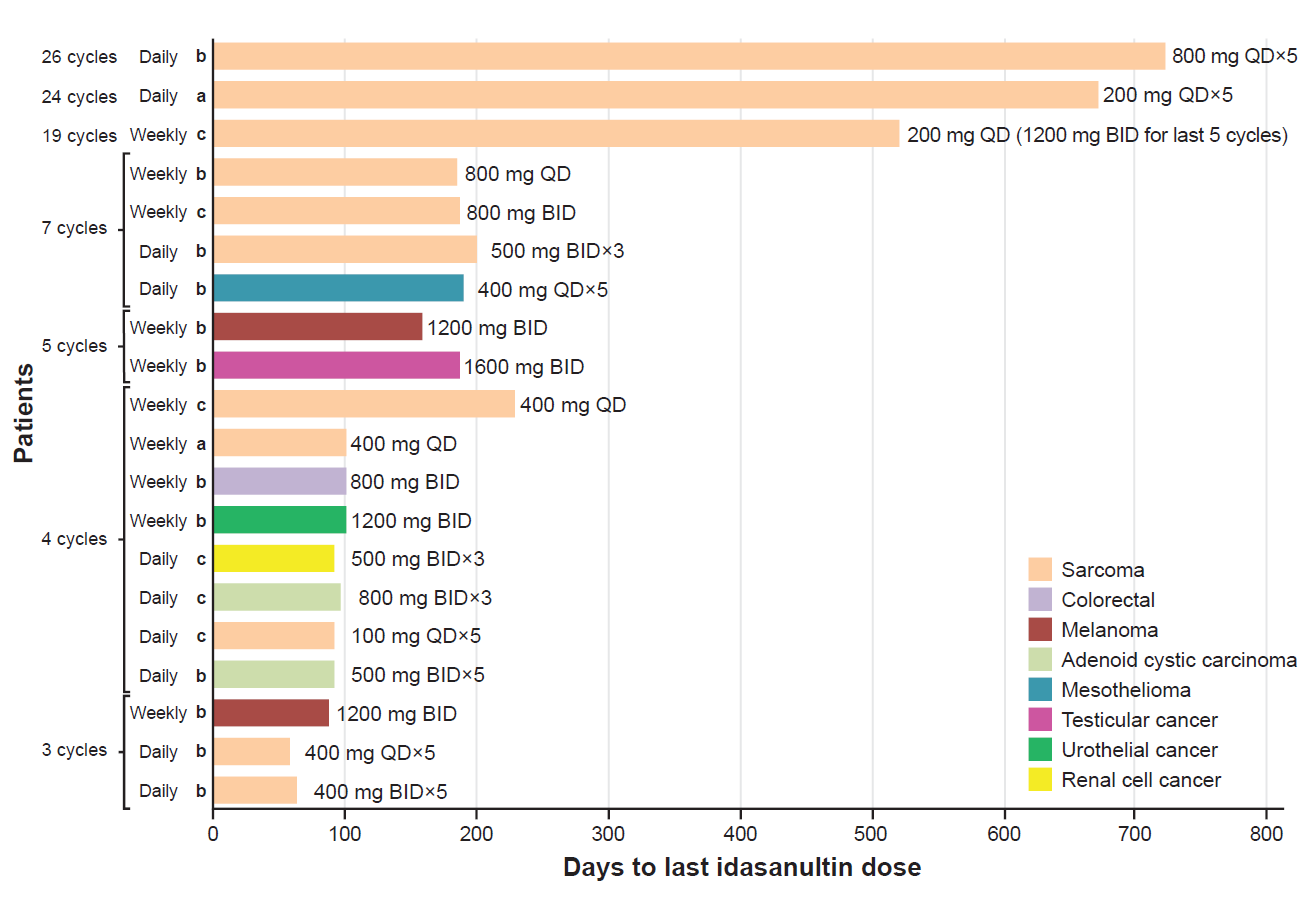


**Fig. S2** SUV_max_ change from baseline during cycle 1 as measured by positron emission tomography with ^18^fluorothymidine. Horizontal dotted line indicates PPR (−25% SUV_max_ change from baseline). Avg, average; BID, twice daily; PPR, partial proliferative response; QD, once daily; SUV_max_, maximum standardized uptake value. ^a^ *TP53* mutant; ^b^ *TP53* wild type.


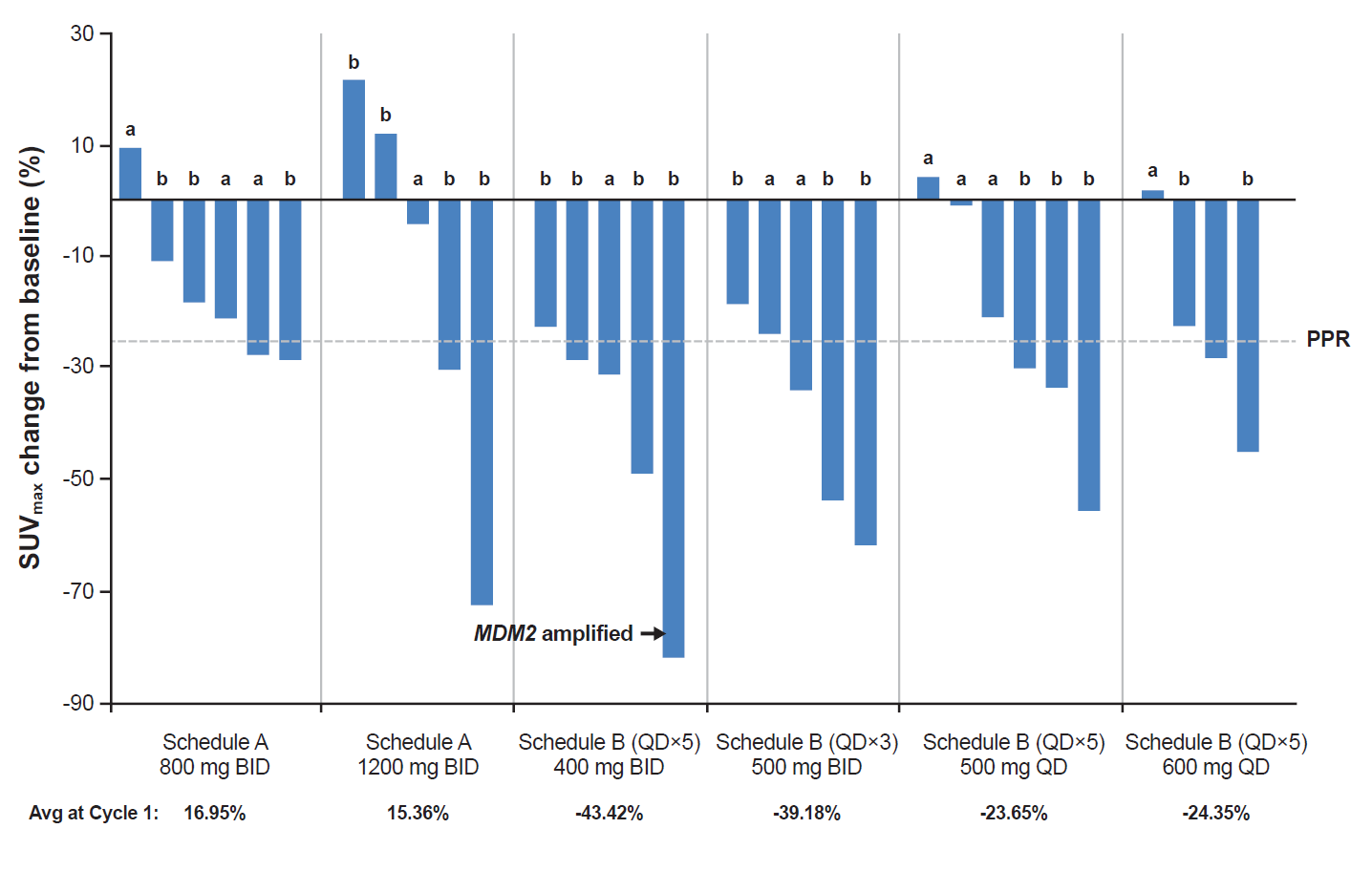

Supplement: Supplementary file 1 — Supplementary file1 (DOCX 221 KB) [file 10637_2021_1141_MOESM1_ESM.docx]
