# Supplementary material for: Phase I study of daily and weekly regimens of the orally administered MDM2 antagonist idasanutlin in patients with advanced tumors
Source: Invest New Drugs. 2021 Jun 28;39(6):1587–97. doi: 10.1007/s10637-021-01141-2 (PMC8541972; doi:10.1007/s10637-021-01141-2)
Supplement: Supplementary file 2 — Supplementary file2 (DOCX 41 KB) [file 10637_2021_1141_MOESM2_ESM.docx]

**SUPPLEMENTARY INFORMATION**

**Phase I Study of Daily and Weekly Regimens of the Orally Administered MDM2 Antagonist Idasanutlin in Patients with Advanced Tumors**

Antoine Italiano,**^1,2,3^** Wilson H. Miller, Jr.,**^4^** Jean-Yves Blay,**^5^** Jourik A. Gietema,**^5^** Yung-Jue Bang,**^7^** Linda R. Mileshkin,**^8^** Hal W. Hirte,**^9^** Brian Higgins,**^10^** Steven Blotner,**^10^** Gwen L. Nichols,**^10^** Lin Chi Chen,**^10^** Claire Petry,^11^ Qi Joy Yang,^12^ Christophe Schmitt,**^11^** Candice Jamois,**^11^** Lillian L. Siu**^13^**

**^1^**Institut Bergonié, Bordeaux, France; **^2^**Gustave Roussy, Villejuif, France; ^3^Faculty of Medicine, University of Bordeaux, Bordeaux; ^4^Segal Cancer Center, Jewish General Hospital, McGill University, Montreal, QC, Canada; **^5^**Centre Léon Bérard, Lyon, France; **^6^**University Medical Center Groningen, Groningen, Netherlands; ^7^Seoul National University College of Medicine, Seoul, South Korea; **^8^**Peter MacCallum Cancer Center, Melbourne, Australia; **^9^**Juravinski Cancer Centre, Hamilton, ON, Canada; **^10^**Hoffmann-La Roche, Roche Innovation Center, New York, NY, USA; **^11^**Roche Innovation Center, Basel, Switzerland; ^12^Certara, Montréal, QC, Canada; **^13^**Princess Margaret Cancer Centre, Toronto, ON, Canada

**Correspondence to:** Dr. Lillian L. Siu, Princess Margaret Cancer Centre, University of Toronto, Toronto, ON M5G 1Z5, Canada. Tel: 416-946-2911; Fax: 416-946-4467; email: lillian.siu@uhn.ca

**SUPPLEMENTARY METHODS**

**Dose-limiting toxicity (DLT)**

DLTs, excluding cytopenias, were assessed during the first treatment cycle (28 days). Cytopenias were followed to nadir (or onset of grade 4 toxicity) for consideration of DLT. The following events were considered DLT:

- Any non-hematologic toxicity considered probably related to study drug, and not attributable to underlying disease progression, of ≥ grade 3 severity (except for grade 3 fatigue, anorexia, and alopecia)
- Nausea/vomiting and/or diarrhea were considered DLT only if they reached ≥ grade 3 severity despite adequate supportive care measures (> 24 hours of treatment) and were considered probably related to study drug
- Grade 4 neutropenia lasting ≥ 7 days (treatment with cytokines was acceptable if patient had grade 4 neutropenia)
- Febrile neutropenia: absolute neutrophil count < 1.0 × 10^9^/L and temperature ≥ 38.5° C
- Grade 3 elevated aspartate aminotransferase or alanine aminotransferase lasting for > 7 days, or grade 4 lasting for any duration of time
- Thrombocytopenia grade 4 (i.e., platelet count < 25.0 × 10^9^/L) or any thrombocytopenia requiring platelet transfusion
- Due to the potential for delayed thrombocytopenia, any patients with grade 2 or higher thrombocytopenia that was not beginning to recover by day 28 was followed to platelet nadir (or grade 4 thrombocytopenia) prior to the dosing decision for the subsequent cohort. This nadir value was used to determine if the thrombocytopenia met DLT criteria. Prolonged platelet nadir (delay > 14 days in initial platelet recovery) or delay in starting cycle 2 more than 28 days (after day 56) due to slow recovery from thrombocytopenia (to platelet count > 100 × 10^9^/L) was also considered dose limiting and was taken into account for subsequent dosing decisions

**Assessment of Food Effects**

To assess the effect of food on the PK of idasanutlin, 10 patients were given 3 single 800-mg doses 1 week apart (i.e., a QW×3 regimen) under either fed or fasted dosing conditions in a randomized, 3-way, partial replicate, crossover design. Patients received idasanutlin under fasted and fed conditions in the first 2 weeks, plus a replicate of fasted or fed dosing in the third week. Fed dosing conditions employed a high fat, high calorie meal, with fat providing approximately 50% of the 1000 kilocalories total caloric content of the meal.

**Pharmacokinetics and Blood Sampling Schedule**

Blood samples for PK determination of idasanutlin plasma concentrations were collected at the following prespecified time points (Online Resource Table S1). For patients receiving the weekly schedule, samples were collected on day 1 pre-dose and at 1, 2, 3, 4, 6, 8, and 12 hours post-dose; samples were also collected at the following time points post-dose: 24, 48, 72 or 96, 120 or 144, and 168 hours. On day 15 (last dosing day in the cycle), the same timepoints were analyzed as on day 1 post-dose (i.e., 0–12 hours).

For the daily schedule, samples were collected pre-dose and on day 1 post-dose at 1, 2, 3, 4, 6, 8, 12, and 24 hours. On days 3 or 5 (or last dosing day in cycle), samples were taken at pre-dose and at 1, 2, 3, 4, 6, 8, 12, 24, 48, 72 or 96, 120 or 144, and 168 hours.

Plasma concentrations of idasanutlin were measured using a validated liquid chromatography/tandem mass spectrometry (LC-MS/MS) method. Non-compartmental PK analysis (including derivation of AUC_0-12h_, AUC_0-24h_, AUC_last,_ AUC_inf_, C_max_, t_max_, and t_½_) was performed using WinNonlin v5.2 or higher (Certara). The food effect on idasanutlin exposure was evaluated using analysis of variance.

**Pharmacodynamic (PD) and Biomarker Assessments**

Serum concentrations of the p53 activation marker macrophage inhibitory cytokine-1 (MIC-1) were analyzed by a solid-phase sandwich ELISA (R&D Systems) at the same time points used for PK assessment. Archival and pre-dose tumor samples (for biomarker and apoptosis cohorts) were assessed for *TP53* mutation status using a microarray-based AmpliChip p53 Test (Roche Molecular Systems).

Patients enrolled in the biomarker cohorts were, where feasible, evaluated by positron emission tomography with ^18^fluorothymidine ([^18^F]-FLT-PET) following treatment to assess changes in tumor proliferation rates with idasanutlin treatment. Standardized uptake values (SUVs) were determined from [^18^F]-FLT-PET imaging scans. Patients whose tumors were not [^18^F]-FLT avid upon screening did not undergo further [^18^F]-FLT-PET imaging.

**Population PK/PD Analysis**

A population PK model developed with NONMEM version 7.3 on data from 623 patients (398 with relapsed/refractory acute myeloid leukemia (AML), 9 unfit patients with AML (> 60 years old), and 216 patients with solid tumors; (NCT01773408, NCT02545283, NCT01901172, NCT03362723, NCT03362723, NCT02828930, NCT01462175) was used to derive the cumulative idasanutlin exposure over 28 days of treatment (i.e., AUC_0-28d_) in patients from this study.

The relationships between exposure to idasanutlin over 28 days and the risk of myelosuppressive events (i.e., occurrence of grade ≥ 3 thrombocytopenia and neutropenia) was characterized using logistic regression models. Logistic regression curves were fitted to the exposure-safety data. A confidence interval (CI), defined as the 5th and 95th percentiles of the model predictions, was defined around each logistic regression using software R.

As idasanutlin indirectly stimulates the production of MIC-1 via blocking interaction between MDM2 and p53, an indirect PK/MIC-1 model developed with NONMEM version 7.3 on data from 99 with solid tumors (NCT01462175) was used to simulate the expected MIC-1 release following a daily or weekly regimen with 50, 100, and 150 mg of idasanutlin.

**SUPPLEMENTARY TABLES**

**Table S1** Pharmacokinetic assessments. QD, once daily; QW, once weekly.

**A.** QW×3 schedule

| **Study day** | **Hours post-dosing** |
| --- | --- |
| 1 | 0 pre-dose |
|  | 1 |
|  | 2 |
|  | 3 |
|  | 4 |
|  | 6 |
|  | 8 |
|  | 12 ± 2 |
| 2 | 24 |
| 3 | 48 |
| 4 or 5 | 72 or 96 |
| 6 or 7 | 120 or 144 |
| 8 | 0 pre-dose (or 168) |
| 15 or final dosing day | 0 pre-dose |
|  | 1 |
|  | 2 |
|  | 3 |
|  | 4 |
|  | 6 |
|  | 8 |
|  | 12 ± 2 |
| 16 or final dosing day (+1 day) | 24 |
| 17 or final dosing day (+2 days) | 48 |
| 18 or 19 or final dosing day (+3 or 4 days) | 72 or 96 |
| 20 or 21 or final dosing day (+5 or 6 days) | 120 or 144 |
| 22 or final dosing day (+7 days) | 168 |

**B.** QD schedules

| **Study Day** | **Hours post-dosing** |
| --- | --- |
| 1 | 0 pre-dose |
|  | 1 |
|  | 2 |
|  | 3 |
|  | 4 |
|  | 6 |
|  | 8 |
|  | 12 ± 2 |
| 2 | 24 pre-dose |
| 5 or final dosing day | 0 pre-dose |
|  | 1 |
|  | 2 |
|  | 3 |
|  | 4 |
|  | 6 |
|  | 8 |
|  | 12 ± 2 |
| 6 or final dosing day (+1 day) | 24 |
| 7 or final dosing day (+2 days) | 48 |
| 8 or 9 or final dosing day (+3 or 4 days) | 72 or 96 |
| 10 or 11 or final dosing day (+5 or 6 days) | 120 or 144 |
| 12 or final dosing day | 168 |
| Biopsy day | 0 pre-dose |

**Table S2** Patient demographics and baseline tumor characteristics

|  | **Weekly dosing (QW×3)^a^**  (n = 36) | **Daily dosing** | | **Food effect cohort**  (n = 10) | **Apoptosis cohort**  (n = 4) | **Total**  (n = 99) |
| --- | --- | --- | --- | --- | --- | --- |
|  |  | **QD×3**  (n = 15) | **QD×5**  (n = 34) |  |  |  |
| **Sex, n (%)**  Male  Female | 24 (66.7)  12 (33.3) | 9 (60.0)  6 (40.0) | 18 (52.9)  16 (47.1) | 7 (70.0)  3 (30.0) | 3 (75.0)  1 (25.0) | 61 (61.6)  38 (38.4) |
| **Race, n (%)**  White  Black  Asian | 32 (88.9)  0  4 (11.1) | 14 (93.3)  0  1 (6.7) | 25 (73.5)  0  9 (26.5) | 9 (90.0)  1 (10.0)  0 | 4 (100.0)  0  0 | 84 (84.8)  1 (1.0)  14 (14.1) |
| **Age, y**  Mean (SD)  Median (range) | 53.4 (11.2)  54.5 (23-74) | 54.7 (15.1)  58.0 (30-76) | 55.0 (12.1)  56.5 (30-74) | 58.2 (5.1)  57.5 (48-66) | 58.0 (20.9)  67.0 (27-71) | 54.8 (12.0)  57.0 (23-76) |
| **BMI, kg/m^2^**  Mean  Median (range) | 25.4 (6.2)  24.2 (17.3-47.6) | 24.9 (5.9)  23.1 (19.6-42.7) | 24.5 (4.8)  23.4 (17.7-37.6) | 25.2 (4.2)  24.4 (20.0-32.0) | 22.4 (2.3)  22.8 (19.2-24.6) | 24.8 (5.4)  23.4 (17.3- 47.6) |
| **ECOG performance status, n (%)**  0  1 | 10 (28.6)  25 (71.4) | 3 (20.0)  12 (80.0) | 14 (41.2)  20 (58.8) | 2 (20.0)  8 (80.0) | 1 (25.0)  3 (75.0) | 30 (30.6)  68 (69.4) |
| **Most common tumor types (≥ 2 patients total), n (%)**  Sarcomas  Colorectal  Melanoma  Urothelial  Adenoid cystic carcinoma  Diffuse large B-cell lymphoma  Mesothelioma  Gastric  NSCLC (adenocarcinoma)  Esophageal  Cervical | 8 (22.2)  5 (13.9)  2 (5.6)  1 (2.8)  0  0  1 (2.8)  1 (2.8)  1 (2.8)  1 (2.8)  2 (5.6) | 4 (26.7)  1 (6.7)  0  2 (13.3)  1 (6.7)  1 (6.7)  0  1 (6.7)  0  0  0 | 10 (29.4)  3 (8.8)  0  1 (2.9)  1 (2.9)  1 (2.9)  1 (2.9)  0  0  1 (2.9)  0 | 0  3 (30.0)  0  1 (10.0)  0  0  0  0  1 (10.0)  0  0 | 1 (25.0)  0  0  0  0  0  0  0  0  0  0 | 23 (23.2)  12 (12.1)  2 (2.0)  5 (5.1)  2 (2.0)  2 (2.0)  2 (2.0)  2 (2.0)  2 (2.0)  2 (2.0)  2 (2.0) |
| **No. of previous anti-cancer regimens, n (%)**  ≤ 2  3–4  5–6  ≥ 7 | 12 (33.3)  15 (41.7)  6 (5.6)  3 (8.3) | 2 (13.3)  7 (46.7)  3 (20.0)  3 (20.0) | 15 (44.1)  8 (23.5)  7 (22.6)  1 (3.2) | 3 (30.0)  4 (40.0)  1 (10.0)  2 (20.0) | 4 (100.0)  0  0  0 | 36 (36.4)  34 (34.3)  17 (17.2)  9 (9.1) |
| ***TP53* mutation status^a^**  Archival tissue sample  Wild type  Mutant  Pre-dose tissue sample  Wild type  Mutant | 17/21 (80.9)  4/21 (19.0)  6/10 (60.0)  4/10 (40.0) | 6/10 (60.0)  4/10 (40.0)  4/5 (80.0)  1/5 (20.0) | 13/20 (65.0)  7/20 (35.0)  9/14 (64.3)  5/14 (35.7) | 3/7 (42.9)  4/7 (57.1)  0/0  0/0 | 0/0  0/0  1/3 (33.3)  2/3 (66.7) | 39/58 (67.2)  19/58 (32.8)  20/32 (62.5)  12/32 (37.5) |

BMI, body mass index; ECOG, Eastern Cooperative Oncology Group; NSCLC, non-small cell lung cancer; QD, once daily; QW, once weekly; SD, standard deviation.

^a^ In cases of discordance between archival and pre-dose samples for an individual patient, the patient was always considered to have mutant status if either sample showed a mutation.

**Table S3** Study drug exposure

|  | **Weekly dosing (QW×3)^a^**  (n = 36) | **Daily dosing^b^** | | **Food effect cohort**  (n = 10) | **Apoptosis cohort**  (n = 4) | **Total**  (n = 99) |
| --- | --- | --- | --- | --- | --- | --- |
|  |  | **QD×3**  (n = 15) | **QD×5**  (n = 34) |  |  |  |
| **Treatment cycles per patient, n (%)** | | | | | | |
| 1 | 9 (25.0) | 6 (40.0) | 13 (38.2) | 3 (30.0) | 1 (25.0) | 32 (32.3) |
| 2 | 17 (47.2) | 6 (40.0) | 14 (41.2) | 7 (70.0) | 2 (50.0) | 46 (46.5) |
| 3–4 | 4 (11.1) | 2 (13.3) | 4 (11.8) | 0 | 1 (25.0) | 11 (11.1) |
| ≥ 5 | 6 (16.7) | 1 (6.7) | 3 (8.8) | 0 | 0 | 10 (10.1) |
| **Duration of treatment** | | | | | | |
| Median (range), days | 43.0 (1–520) | 31.0 (3–204) | 33.0 (2–726) | 40.0 (15–43) | 33.0 (5–122) | 36.0 (1–726) |
| Mean (SD), days | 73.4 (96.3) | 43.2 (54.7) | 74.2 (162.8) | 33.3 (12.9) | 48.3 (50.9) | 64.0 (114.0) |
| 0–91 days, n (%) | 27 (75.0) | 13 (86.7) | 31 (91.2) | 19 (100.0) | 3 (75.0) | 84 (84.8) |
| 92–183 days, n (%) | 4 (11.1) | 1 (6.7) | 0 | 0 | 1 (25.0) | 6 (6.1) |
| 184–274 days, n (%) | 4 (11.1) | 1 (6.7) | 1 (2.9) | 0 | 0 | 6 (6.1) |
| > 365 days, n (%) | 1 (2.8) | 0 | 2 (5.9) | 0 | 0 | 3 (3.0) |
| **No. of doses** | | | | | | |
| Median (range) | 10.5 (2–72) | 9.0 (6–42) | 10.0 (1–130) | 7.5 (3–9) | 10.0 (5–18) | 10.0 (1–130) |
| Mean (SD) | 13.4 (13.0) | 12.3 (9.9) | 19.1 (28.7) | 6.5 (2.7) | 10.8 (5.4) | 19.2 (10.0) |
| Total cumulative dose, median (range), mg | 9600  (600–44400) | 4800  (3000–21000) | 4450  (500–87000) | 6000  (2400–7200) | 5000  (2500–7750) | 6000  (500–87000) |

SD, standard deviation; QD, once daily; QW, once weekly.

^a^ Patients treated on the weekly schedule, including biomarker imaging cohorts, except those enrolled in the food effect sub-study.

^b^ Patients treated on daily schedules (QD×3 or QD×5), including biomarker imaging cohorts, except those enrolled in the apoptosis imaging cohort.

**Table S4** Most common DLTs by dosing schedule in the dose-escalation cohorts^a^

| **DLT, n (%)** | **Weekly dosing**  (n = 36) | **Daily dosing** | |
| --- | --- | --- | --- |
|  |  | **QD×3**  (n = 15) | **QD×5**  (n = 34) |
| Total no. of patients with ≥ 1 DLT, n (%) | 3 (8.3) | 6 (40.0) | 11 (32.4) |
| Diarrhea | 0 | 0 | 1 (2.9) |
| Febrile neutropenia | 0 | 1 (6.7) | 2 (5.9) |
| Nausea | 2 (5.6) | 0 | 0 |
| Neutropenia | 0 | 1 (6.7) | 4 (11.8) |
| Pancytopenia | 0 | 1 (6.7) | 0 |
| Thrombocytopenia^b^ | 1 (2.8) | 5 (33.3) | 10 (29.4) |
| Vomiting | 1 (2.8) | 0 | 0 |

DLT, dose-limiting toxicity; QD, once daily.

^a^ Includes the 85 response-evaluable patients.

^b^ Includes decreased platelet count.

**Table S5** Summary of related adverse events (any grade; overall incidence ≥ 10%) by Medical Dictionary for Regulatory Activities (version 17.0) preferred terms

|  | **Weekly dosing (QWx3)** | | | **Daily dosing** | | | | **Apoptosis**  **QD×5** | **Total** |
| --- | --- | --- | --- | --- | --- | --- | --- | --- | --- |
|  |  |  |  | **QD×3** | | **QD×5** | |  |  |
|  | **Dose escalation**  **(n = 23)** | **Biomarker**  **(n = 13)** | **Food Effect**  **(n = 10)** | **Dose Escalation**  **(n = 9)** | **Biomarker**  **(n = 6)** | **Dose Escalation**  **(n = 16)** | **Biomarker**  **(n = 18)** | **Apoptosis**  **(n = 4)** | **Total**  **(N = 99)** |
| Total no. of patients with  ≥ 1 related AE, n (%)^a^ | 23 (100.0) | 13 (100.0) | 10 (100.0) | 9 (100.0) | 6 (100.0) | 16 (100.0) | 18 (100.0) | 4 (100.0) | 99 (100.0) |
| Overall total no. of related AEs | 302 | 87 | 46 | 67 | 76 | 192 | 185 | 21 | 976 |
| Diarrhea, n (%) | 19 (82.6) | 12 (92.3) | 6 (60.0) | 6 (66.7) | 5 (83.3) | 11 (68.8) | 14 (77.8) | 1 (25.0) | 74 (74.7) |
| Nausea, n (%) | 18 (78.3) | 11 (84.6) | 6 (60.0) | 5 (55.6) | 5 (83.3) | 10 (62.5) | 15 (83.3) | 1 (25.0) | 71 (71.7) |
| Vomiting, n (%) | 13 (56.5) | 5 (38.5) | 4 (40.0) | 6 (66.7) | 4 (66.7) | 10 (62.5) | 7 (38.9) | 1 (25.0) | 50 (50.5) |
| Decreased appetite, n (%) | 10 (43.5) | 4 (30.8) | 2 (20.0) | 5 (55.6) | 3 (50.0) | 8 (50.0) | 9 (50.0) | 2 (50.0) | 43 (43.4) |
| Thrombocytopenia, n (%) | 6 (26.1) | 5 (38.5) | 2 (20.0) | 3 (33.3) | 5 (83.3) | 5 (31.3) | 10 (55.6) | 3 (75.0) | 39 (39.4)^b^ |
| Fatigue, n (%) | 5 (21.7) | 5 (38.5) | 1 (10.0) | 0 | 4 (66.7) | 6 (37.5) | 6 (33.3) | 1 (25.0) | 28 (28.3) |
| Anemia, n (%) | 4 (17.4) | 1 (7.7) | 1 (10.0) | 5 (55.6) | 3 (50.0) | 1 (6.3) | 5 (27.8) | 2 (50.0) | 22 (22.2) |
| Asthenia, n (%) | 7 (30.4) | 1 (7.7) | 2 (20.0) | 7 (77.8) | 0 | 0 | 4 (22.2) | 0 | 21 (21.2) |
| Neutropenia, n (%) | 3 (13.0) | 2 (15.4) | 0 | 3 (33.3) | 1 (16.7) | 2 (12.5) | 7 (38.9) | 0 | 18 (18.2)^c^ |
| Dysgeusia, n (%) | 4 (17.4) | 3 (23.1) | 1 (10.0) | 0 | 4 (66.7) | 2 (12.5) | 2 (11.1) | 1 (25.0) | 17 (17.2) |
| Headache, n (%) | 7 (30.4) | 1 (7.7) | 1 (10.0) | 0 | 0 | 3 (18.8) | 2 (11.1) | 0 | 14 (14.1) |
| Pyrexia, n (%) | 3 (13.0) | 0 | 1 (10.0) | 2 (22.2) | 0 | 1 (6.3) | 3 (16.7) | 0 | 10 (10.1) |

AE, adverse event; QD, once daily; QW, once weekly. Multiple occurrences of the same adverse event in one individual counted only once.

^a^ Relationship to study drug as determined by investigator to be either remote, possible, or probable.

^b^ Additional 5 patients (5.1%) reported with platelet count decreased.

^c^ Additional 5 patients (5.1%) reported with neutrophil count decreased.

**Table S6** Serious adverse events by Medical Dictionary for Regulatory Activities (version 17.0) preferred terms

|  | **Weekly dosing (QW×3)** | | | **Daily dosing** | | | | **Apoptosis**  **QD×5** | **Total** | |
| --- | --- | --- | --- | --- | --- | --- | --- | --- | --- | --- |
|  |  |  |  | **QD×3** | | **QD×5** | |  |  |  |
| **Cohort** | **Dose escalation**  **(n = 23)** | **Biomarker**  **(n = 13)** | **Food effect**  **(n = 10)** | **Dose escalation**  **(n = 9)** | **Biomarker**  **(n = 6)** | **Dose escalation**  **(n = 16)** | **Biomarker**  **(n = 18)** | **Apoptosis**  **(n = 4)** | **Total**  **(N = 99)** | |
| Total no. of patients with ≥ 1 SAE, n (%) | 8 (34.8) | 1 (7.7) | 2 (20.0) | 3 (33.3) | 4 (66.7) | 5 (31.3) | 8 (44.4) | 1 (25.0) | 32 (32.3) | |
| Overall total no. of SAEs | 9 | 1 | 2 | 5 | 7 | 10 | 17 | 2 | 53 | |
| Thrombocytopenia, n (%) | 1 (4.3) | 1 (7.7) | 0 | 2 (22.2) | 0 | 3 (18.8) | 4 (22.2) | 1 (25.0) | 12 (12.1)^a^ | |
| Febrile neutropenia, n (%) | 0 | 0 | 0 | 0 | 1 (16.7) | 1 (6.3) | 3 (16.7) | 0 | 5 (5.1) | |
| Anemia, n (%) | 1 (4.3) | 0 | 1 (10.0) | 1 (11.1) | 0 | 0 | 0 | 0 | 3 (3.0) | |
| Neutropenia, n (%) | 1 (4.3) | 0 | 0 | 0 | 0 | 1 (6.3) | 1 (5.6) | 0 | 3 (3.0)^b^ | |
| Leukopenia, n (%) | 0 | 0 | 0 | 0 | 1 (16.7) | 0 | 0 | 1 (25.0) | 2 (2.0)^c^ | |
| Total no. of patients with  ≥ 1 treatment-related SAE, n (%) | 3 (13.0) | 1 (7.7) | 0 | 3 (33.3) | 4 (66.7) | 5 (31.3) | 8 (44.4) | 1 (25.0) | 25 (25.3) | |
| Overall total no. of treatment-related SAEs | 3 | 1 | 0 | 5 | 5 | 5 | 15 | 2 | 36 | |
| Thrombocytopenia, n (%) | 1 (4.3) | 1 (7.7) | 0 | 2 (22.2) | 0 | 3 (18.8) | 4 (22.2) | 1 (25.0) | 12 (12.1) | |
| Febrile neutropenia, n (%) | 0 | 0 | 0 | 0 | 1 (16.7) | 1 (6.3) | 3 (16.7) | 0 | 5 (5.1) | |
| Anemia, n (%) | 1 (4.3) | 0 | 0 | 1 (11.1) | 0 | 0 | 0 | 0 | 2 (2.0) | |
| Neutropenia, n (%) | 0 | 0 | 0 | 0 | 0 | 1 (6.3) | 1 (5.6) | 0 | 2 (2.0) | |
| Leukopenia, n (%) | 0 | 0 | 0 | 0 | 1 (16.7) | 0 | 0 | 1 (25.0) | 2 (2.0) | |
| QD, once daily; QW, once weekly; SAE, serious adverse event. Patients could have ≥ 1 adverse event, and multiple occurrences of the same event were counted only once.  ^a^ Additional 2 patients (2.0%) reported with platelet count decreased.  ^b^ Additional 2 patients (2.0%) reported with neutrophil count decreased.  ^c^ Additional 1 patient (1.0%) reported with white blood cell count decreased. | | | | | | | | | |  |

**Table S7** Effect of food on single-dose idasanutlin pharmacokinetics

| **PK parameter** | **Treatment** | **Geometric least squares mean** | **Ratio**  **(fed/fasted)** | **90% CI** |
| --- | --- | --- | --- | --- |
| AUC_inf obs_, h•ng/mL | Fed 800 mg  Fasted 800 mg | 135,131  94,637 | 1.428 | 0.938, 2.173 |
| AUC_last_, h•ng/mL | Fed 800 mg  Fasted 800 mg | 127,301  91,523 | 1.391 | 0.915, 2.114 |
| C_max_, ng/mL | Fed 800 mg  Fasted 800 mg | 2958  2600 | 1.137 | 0.736, 1.758 |
| T_max_, h | Fed 800 mg  Fasted 800 mg | 8.49  5.95 | 1.427 | 1.017, 2.002 |
| t_1/2_, h | Fed 800 mg  Fasted 800 mg | 31.23  29.14 | 1.072 | 0.809, 1.420 |

AUC_inf obs_, area under the curve extrapolated to infinity based on the last observed concentration; AUC_last_, area under the curve from 0 to the time of the last measurable concentration; C_max_, maximum plasma concentration; CI, confidence interval; PK, pharmacokinetics; t_1/2_, half-life; T_max_, time to maximum concentration. One pair of t_½_ and AUC was excluded as outliers (inappropriate determination of t_½_).
